# Effect of DACH1 on proliferation and invasion of laryngeal squamous cell carcinoma

**DOI:** 10.1186/s13005-018-0177-1

**Published:** 2018-09-27

**Authors:** Jiarui Zhang, Xiuxia Ren, Bo Wang, Jing Cao, Linli Tian, Ming Liu

**Affiliations:** Department of Otorhinolaryngology, Head and Neck Surgery, Second Affiliated Hospital, Harbin Medical University, Harbin, 150081 China

**Keywords:** LSCC, DACH1, Proliferation, Invasion

## Abstract

**Background:**

To investigate the effect of DACH1 over-expression on proliferation and invasion of laryngeal squamous cell carcinoma (LSCC).

**Methods:**

The 120 cases of LSCC tumors and 114 adjacent non-neoplastic tissues were collected to detect the expression of DACH1 by immunohistochemistry. The changes of DACH1 expression from each group were assessed and correlated to the clinical parameters of the patients. Plasmid-DACH1 was transfected into Hep-2 cells to up-regulate the expression of DACH1C. Real-time PCR, Western blot, CCK8 and transwell assay were used to verify the cell proliferation and invasion after plasmid-DACH1 transfection.

**Results:**

The results indicated that DACH1 was downregulated in LSCC tissues as compared to corresponding adjacent non-neoplastic tissues. Decreased expression of DACH1 was found in the tumors upraglottic tumor, lymph node metastases, T3–4 stage and advanced clinical stage. In Hep-2 cells, transfection with plasmid-DACH1 could suppress cell proliferation, invasion and induce G1 phase extension in cell cycle.

**Conclusions:**

DACH1 may act as a tumor suppressor gene and could be a potential target for therapeutic intervention of LSCC.

## Background

One of the most common head and neck cancers is laryngeal cancer, which is also the third most common otolaryngological cancer [1]. The majority of laryngeal cancers are laryngeal squamous cell carcinomas (LSCC). Despite improvements in diagnostic and therapeutic modalities, there has been no significant improvement in laryngeal cancer survival over the past 20 years [2, 3]. Therefore, new diagnostic and therapeutic targets for LSCC are urgently needed.

The dachshund (DAC) gene was first elucidated in drosophila and isolated as a dominant suppressor of mutation ellipse. DACH1, as a homologous gene in humans, associates with the Retinal Determination Gene Network (RDGN), which includes DAC/DACH, eya/Eya, so/Six, ey and toy [4, 5]. DACH1 is a tumor suppressor gene in many cancers such as colorectal, oral and breast cancers [6–8]. Among the mammals, the expressions of DACH1 target genes could be through DNA-binding transcription factors and DNA-sequence specific binding to Forkhead binding sites [9]. DACH1 was reported to be related to epithelial-mesenchymal transition (EMT): E-cadherin and γ-catrnin that belong to the epithelial protein are down-regulated, while N-cadherin and vimentin that belong to mesenchymal protein are up-regulated [6]. DACH1 can negatively regulate TGF-β and Wnt pathways, repress SNAI1 and CXCL5 signaling [4, 7, 8, 10]. The acetylated carboxyl terminus of DACH1 binds to P53 and enhances its tumor suppressor function in breast cancer [11]. The methylation of DACH1 also promotes the motility and invasion of tumors [8]. Although DACH1 has been studied in many cancers, its role in LSCC remains unknown. Therefore, we examined the in vivo and in vitro relationship between DACH1 expression and LSCC to determine if DACH1 had any anticancer effects in LSCC.

## Methods

### Patients and samples

A total of 120 cases of LSCC tumors and 114 adjacent non-neoplastic tissues were collected from the Department of Pathology in the Second Affiliated Hospital of Harbin Medical University. The LSCC patients were treated by surgery from 2014 to 2016, and no patient received any anticancer treatment before surgery. All patients had no prior history of other cancer and precursor lesions. The fresh tissues were immediately fixed in buffered formalin. We analyzed gender, age, smoking, drinking, T classification, lymph node metastases, primary location and histopathological differentiation, which were obtained from patient records. Smoking and alcohol consumption were calculated according to [12].

### Cell culture and transfection

The human LSCC cell line Hep-2 was purchased from The Cell Bank of Chinese Academy of Science (Shanghai, China). Plasmid-DACH1 was constructed by Origene. Hep-2 cells were cultured in DMEM (Hyclone) supplemented with 10% fetal bovine serum (NQBB) and incubated at 37under humidified atmosphere containing 5% CO_2_. DACH1 plasmid with GFP as a reporter gene was transfected into Hep-2 cells with Lipofectamine 2000 (Invitrogen) according to the manufacturer’s instructions. Hep-2 cells were plated onto 6-well plates (2.5 × 10^5^ cell/well) for a day till they reached 80–85% confluency. The plasmid and Lipofectamine 2000 were each diluted in 250 uL of serum-free OPTI-MEM (Gibco BRL) and incubated for 5 min at room temperature. The diluted plasmid and Lipofectamine 2000 were combined at a 1:2 ratio (3 μg of plasmid with 6 uL of Lipofectamine 2000), mixed gently and incubated for 20 min at room temperature. A total of 500 uL of the mixture was added to each well in a final volume of 2 mL per well. Then the cells were analyzed by Real-time PCR and Western blot.

### Real-time PCR and Western blotting

Total RNA was isolated using Trizol reagent (Invitrogen) according to the manufacturer’s instructions. About 500 ng of total RNA was used to synthesize cDNA according to the manufacturer’s manual (TOYOBO FSQ-301). The primer sequences of DACH1 were designed and synthesized by Invitrogen: Forward primer was 5′-TGCCGCATTCTGTCCCT-3′ and Reverse primer was 5′-GAGTCTGCTCCATGTTGGTTATT-3′. After reverse transcription at 37 °C for 15 min, 50 °C for 5 min and 98 °C for 5 min, Real-time PCR was performed using SYBR-Green Master Mix (TOYOBO QPK-201). Reaction conditions were 95 °C for 60 s, 40 cycles at 95 °C for 15 s, 56 °C for 15 s, and 72 °C for 45 s. Data calculated from the Ct values were normalized to the expression of human β-actin gene, and 2^-△△Ct^ was used to calculate the expression of DACH1. Each sample was run in triplicate.

Untreated Hep-2 cells and GFP-plasmid groups were collected and analyzed by western blot to assess the expression of DACH1. Total protein was extracted from each group. Anti-DACH1 antibody was purchased from Origene. The membrane with anti-DACH1 (diluted 1:5000 in TBST) was incubated overnight at 4 °C, followed by incubation with the secondary antibody for 1 h at room temperature. β-actin was used as a loading control. The signal intensities were determined using Image J program.

### Cell proliferation and invasion assays

Black Hep-2 cells were used as controls for cells transfected with DACH1 plasmid. Cells were plated in 96-well plates at a density of 5 × 10^3^ cells per well. To measure cell proliferation at 24 h, 48 h, 72 h, 10 uL CCK8 reagent was added to cells according to the manufacturer’s protocol, and incubated at 37 °C for 2 h. The absorbance at 450 nm was measured using a micro-well plate reader. Each group had five replicate wells. The percentage rate of cell growth was calculated using the following formula: (mean absorbance of the treatment group/mean absorbance of the control group) × 100.

The invasion ability between plasmid-DACH1 levels and black Hep-2 cells was compared using 24-well Boyden chambers (8 mm pore size) coated with matrigel (BD). A total of 2 × 10^4^ cells in 200 uL serum-free media were resuspended in the upper chambers. The lower chamber contained 600 uL of medium containing 10% fetal bovine serum to serve as a chemoattractant. Cells were incubated for 36 h at 37 °C, then cells in the upper chambers were mechanically removed. The cells that had migrated to the lower chambers were fixed in crystal violet for 10–30 min, and five random fields were observed and counted under microscope at × 200 magnification. The experiments were repeated at least three times.

### Cell cycle analysis

After transfecting with plasmid-DACH1 for 72 h, the cells were washed twice with cold PBS and fixed in 75% ethanol for 24 h. Then the cells were stained as per the manufacturer’s instructions (BD), and cell cycle was analyzed by flow cytometry.

### Immunohistochemistry

The samples were embedded in paraffin and incised into thin sections (4um). Then the sections were dewaxed with xylene and different doses alcohol, and washed three times with distilled water. Antigen retrieval was performed with repair solution according to the manufacturer’s instructions. Slices were infiltrated with 3% H_2_O_2_, put into humidors for 10 min, and washed three times with PBS. Rabbit anti-DACH1 (OriGene) was added and the slices were incubated overnight at 4 °C. Slices were rinsed five times with PBS for five minutes each time, incubated with secondary antibody for 30 min at room temperature, and then washed three times with PBS. The slides were stained with a drop of 3,3-di-aminobenzidine (DAB) and counterstained with hematoxylin for three minutes, dehydrated by alcohol, and sealed by neutral gum. Positive cells showed a brownish color and negative cells were blue.

### DACH1 scores

The DACH1 IHC staining results were evaluated by three independent pathologists. DACH1 expression was observed in the nucleus of cells in five random fields. The staining intensity was evaluated based on a 4-point scale (0, no staining, 1, weak intensity, 2, moderate intensity, and 3, strong intensity). The positive cancer cells were also evaluated on a 5-point scale according to the fraction of stained cells (0, < 1%, 1, 1–10%, 2, 10–50%, 3, 50–80%, 4, 80–100%). The DACH1 expression was calculated as the percentage of positive cells in whole tumor cells. DACH1 low-expression based on the percentage of positive cells was lower than the mean value. The IHS was equal to the staining intensity multiplied by the fraction of stained cells.

### Statistical analysis

The expression of DACH1 was analyzed by Chi-square test. Independent-sample t-test were used to compare the differences between the two groups. *P* < 0.05 was considered to be significant. All statistical calculations were expressed as mean ± SD using the SPSS statistical software. Graphpad Prism 5 was used to create the artworks.

## Results

### DACH1 expression is low in LSCC

**Fig. 1:**
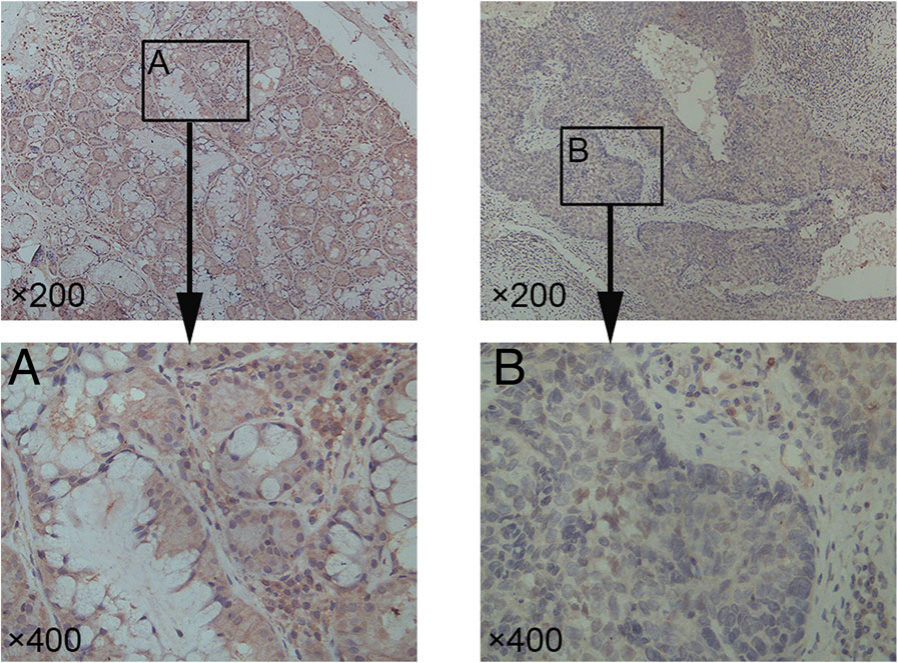
Representative DACH1 expression level in non-neoplastic tissues was lower than that in adjacent LSCC determined by IHC. **a** Representative DACH1 expression level in non-neoplastic tissues (upper images, × 200; lower image, × 400). **b** Representative DACH1 expression level in LSCC(upper images, × 200; lower image, × 400)

IHC staining for DACH1 in 120 LSCC and 114 adjacent non-neoplastic mucosa paraffin specimens showed that the intensity and percentage of stained cells in the former samples were obviously lower than the latter. DACH1 was mainly found in the nucleus (Fig. 1), and its expression was associated with several clinical parameters, including smoking, drinking, T classification, lymph node metastases, primary location and clinical stage in LSCC paraffin specimens (Table 1). The low-expression was related to supraglottic tumors, T3–4 stage, and III-IV clinical stage. DACH1 had no significant difference with age, gender, and differentiation.

**Table 1:** Relationship between DACH1 expression and clinicopathological characteristics in 120 LSCC patients

Clinicopathological characteristics	DACH1	expression	*p*-value
	<mean value%	≥mean value%	
*N* = 120	60 (50.0%)	60 (50.0%)	
Age (mean ± SD; years)			0.239
< 61 ± 8.11	38 (31.7%)	44 (36.7%)	
≥ 61 ± 8.11	22 (18.3%)	16 (13.3%)	
Gender			0.194
Male	32 (26.7%)	39 (32.5%)	
Female	28 (23.3%)	21 (17.5%)	
T classification			0.002
T_1_-T_2_	49 (40.8%)	33 (27.5%)	
T_3_-T_4_	11 (9.2%)	27 (22.5%)	
Lymph node metastases			0.010
Yes	40 (33.3%)	52 (43.3%)	
No	20 (16.7%)	8 (6.7%)	
Primary location			0.006
Supraglottic	41 (34.2%)	26 (21.7%)	
Glottic	19 (15.8%)	34 (28.3%)	
Differentiation			0.099
G1	23 (19.2%)	32 (26.7%)	
G2-G3	37 (30.8%)	28 (23.3%)	
Clinical stage			0.016
I-II	29 (24.2%)	42 (35.0%)	
III-IV	31 (2ss5.8%)	18 (15.0%)	
Smoking			0.002
Never	4 (3.3%)	17 (14.2%)	
Ever	56 (46.7%)	43 (35.8%)	
Drinking			0.039
Never	11 (9.2%)	21 (17.5%)	
Ever	49 (40.8%)	39 (32.5%)	

### DACH1 expression was up-regulated after DACH1 plasmid transfection in Hep-2 cells

**Fig. 2:**
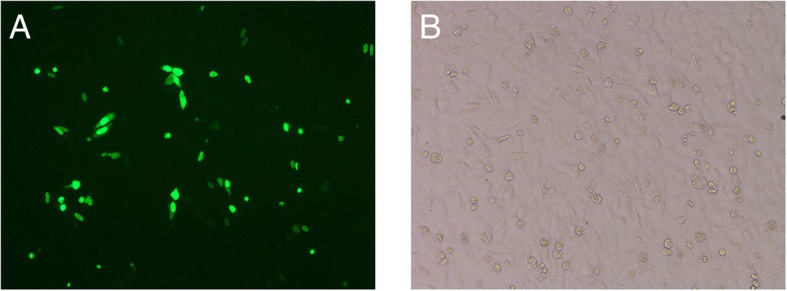
(**a**) GFP plasmid-DACH1 expression in Hep-2 cells after 72 h. **b** No GFP expression in black Hep-2 cells. Fluorescence microscope images (× 200)

**Fig. 3:**
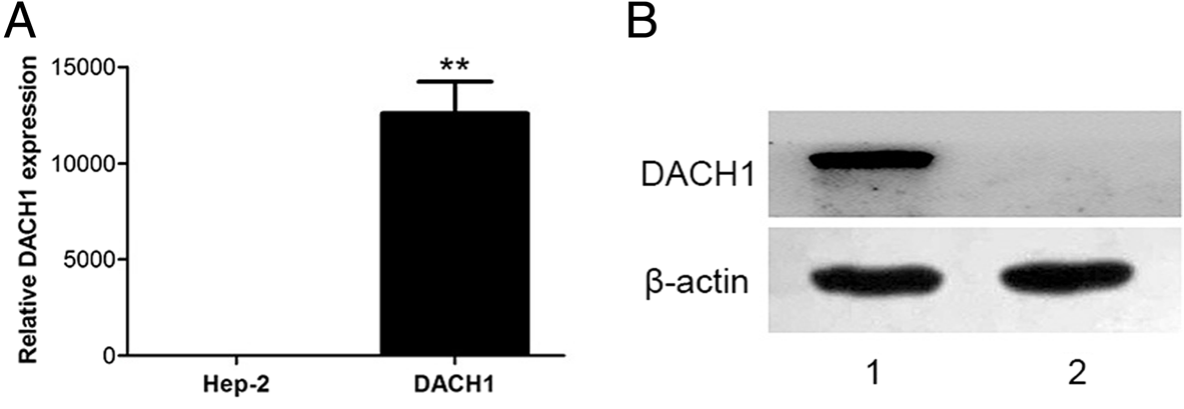
The expression level of DACH1 in two groups. **a** Real-time PCR analysis showed that DACH1 expression was significantly up-regulated after plasmid-DACH1 transfection, *P* < 0.05. **b** Western blot showed the protein level of DACH1 in transfected Hep-2 cells(1), and control Hep-2 cells (2)

Plasmid-DACH1 was transfected in Hep-2 cells to up-regulate the expression of DACH1 in order to further explore the functional roles of DACH1 in LSCC (Fig. 2). Real-time PCR and Western blot were used to verify the translational level after plasmid-DACH1 transfection, which showed that both mRNA (Fig. 3a) and protein (Fig. 3b) levels were higher in the plasmid-DACH1 transfected group than in the uninfected group. These results indicated that DACH1 was effectively expressed after plasmid-DACH1 transfection in Hep-2 cells.

### Overexpression of DACH1 inhibited proliferation and invasion of Hep-2 cells

**Fig. 4:**
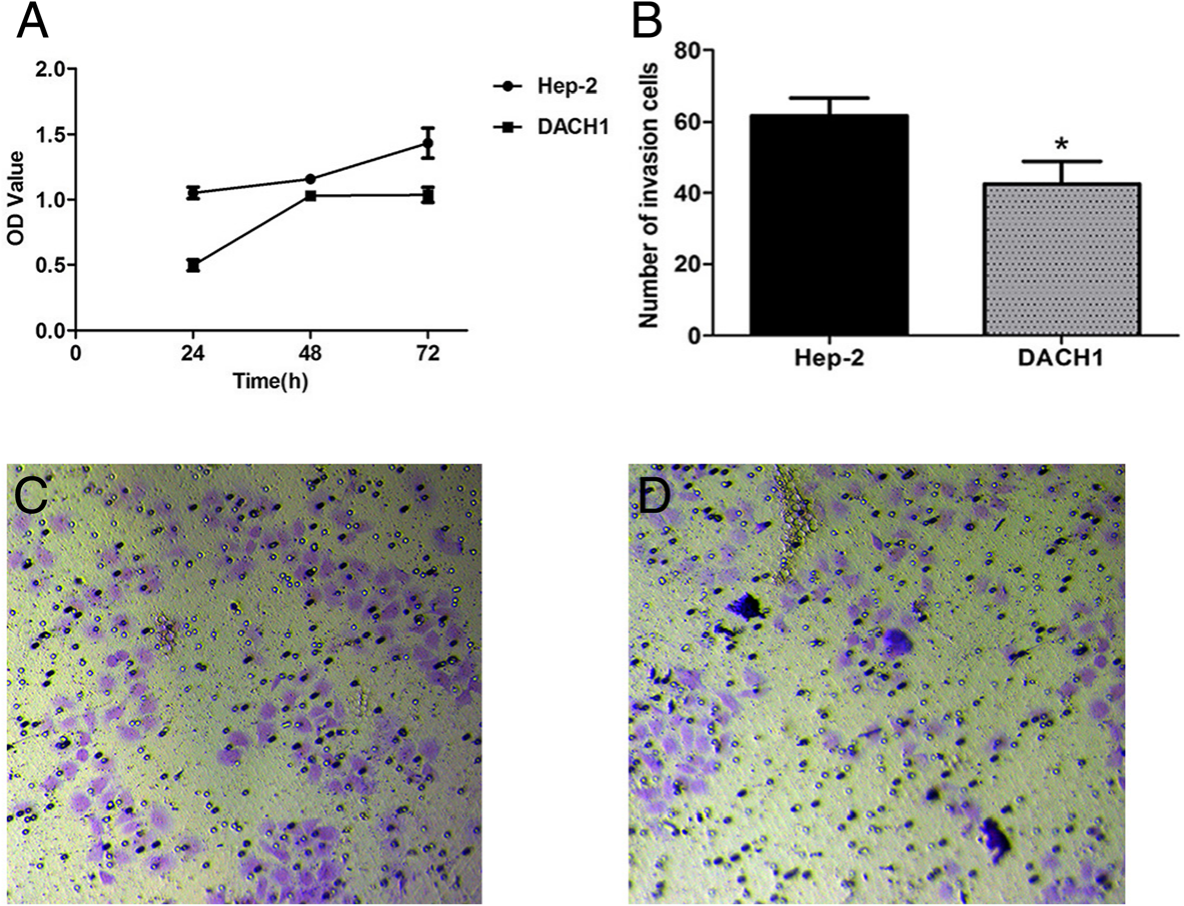
DACH1 inhibited proliferation and invasion of Hep-2 cells. **a** After plasmid-DACH1 transfection, the OD of the transfected group had no obvious increase as compared to the infected group at 72 h, *P* < 0.05. **b** After 72 h transfection, the number of invasive cells in each group. **c** The number of invasive cells in the black Hep-2 cells group. **d** The plasmid-DACH1 group, *P* < 0.05

After transfecting plasmid-DACH1 in Hep-2 cells, we tested its influence on the proliferation and invasion of cells. As shown in Fig. 4a, proliferation of Hep-2 cells transfected with plasmid-DACH1 was effectively inhibited at 72 h. The growth curve was plotted according to OD values. Transwell assay suggested that the invasion of Hep-2 cells was significantly lower in the transfected group than in the control group (Fig. 4b, c and d). The results implied that DACH1 inhibited the proliferation and invasion of Hep-2 cells in vitro. Therefore, DACH1 may play a role in inducing the proliferation and invasion of LSCC.

### Overexpression of DACH1 suppressed cell cycle

**Fig. 5:**
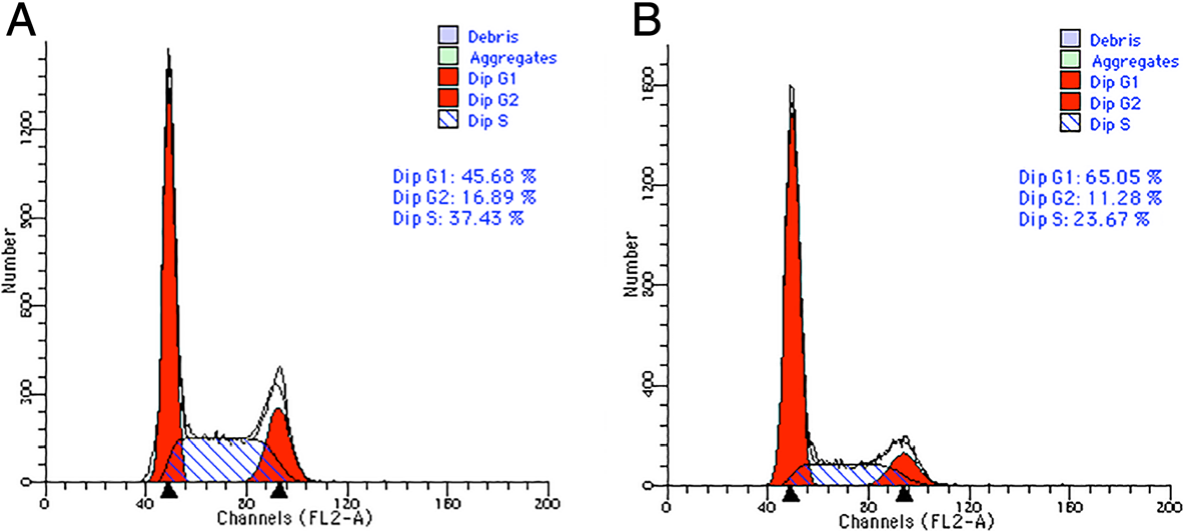
Flow cytometric analysis of the effect of DACH1 on the cell cycle distribution in Hep-2 cells after transfection for 72 h. The G1 phase of black Hep-2 cells is significantly lower than plasmid-DACH1 cells, P < 0.05. **a** Cell cycle analysis of the blank control group Hep-2 cells. **b** Cell cycle analysis of DACH1-transfected Hep-2 cells

We detected cell cycle to study the antiproliferative mechanism of DACH1 and flow cytometry analysis indicated that up-regulation of DACH1 in Hep-2 cells could suppress cell cycle in G1 phase, as compared to the uninfected group, the G1 phase of the plasmid-DACH1 transfected group was markedly prolonged (Fig. 5).

## Discussion

LSCC accounts for a vast proportion of head and neck cancers. In 2011, LSCC accounted for approximately 0.7% of all new cancer diagnoses and about 0.6% of all cancer-related deaths [13]. Metastasis is one of the main reasons for poor prognosis. Studies have shown that EMT may contact with tumor invasion and metastasis [14]. DACH1 could repress EMT by repressing gene transcription in the nucleus and translation in the cytoplasm [15].

DACH1 has been identified in several human cancers as a novel tumor suppressor gene [16–18]. Our study showed that DACH1 was significantly down-regulated in LSCC as compared to non-neoplastic tissues. In addition, it was found that lower expression of DACH1 was closely correlated with supraglottic tumor, lymph node metastases, T3–4 stage and advanced clinical stage. Considering the association of these clinicopathological parameters with the poor prognosis of patients with LSCC, these results imply that DACH1 may play a role in the progression and influence the prognosis in LSCC. To explore the effect of the overexpression of DACH1on the metastasis and progression of LSCC, we examined the alterations in cell growth and behavior following DACH1overexpression in the Hep-2 cells. Elevated expression of DACH1 suppressed proliferation, induced cell cycle arrest.

in the G1 phase, and suppressed cell invasion in the Hep-2 cells. Taken together, these results suggest that DACH1 is a tumor-suppressor in the growth and progression of LSCC. These results were similar to previous studies on the expression of DACH1 in esophageal cancer, hepatocellular carcinoma and colorectal cancer [10, 18, 19]. Smoking and alcohol consumption were risk factors of laryngeal carcinoma, and DACH1 increased the risk of laryngeal carcinoma which is consistent with other study [12].

Patients with relatively low DACH1 expression showed lower 5-year overall survival among lung adenocarcinoma, endometrial cancer and luminal breast cancer [4, 17, 20]. DACH1 may be a prognosis gene in LSCC. However, DACH1 is up-regulated in myeloid leukemia via interaction with HOXA9 and overexpression in ovarian cancer with poor prognosis as promotion sensitivity gene [21, 22]. So, in order to examine how DACH1 suppresses LSCC, we transfected plasmid-DACH1 in Hep-2 cells, and used RT-PCR and Western blot assays to verify its successful expression. The results showed that over-expression of DACH1 suppressed Hep-2 cell growth and invasion, which was in agreement with previous studies that showed that low-expression of DACH1 promoted the activation of MMP-2 and MMP-9 through TGF-β signaling to promote invasion in gastric cancer [23] and high-expression of DACH1 blocked Wnt pathway to suppress viability and invasion in hepatocellular carcinoma [24]. DACH1 was found to be the less frequent methylation in oral carcinoma cell lines as the negative regulator of Wnt pathway [8]. Furthermore, we found that over-expression of DACH1 suppressed the G1 phase to prolong cell cycle. These results indicated that DACH1 may act as an anti-tumor gene in Hep-2 cells. The underlying mechanism needs further investigation.

MiRNA-194 and miRNA-217 are known to target DACH1 in pancreatic ductal adenocarcinoma and breast cancer, respectively. They could block the expression of DACH1, and promote the development of tumors [25, 26]. However, the relationship between these miRNAs and DACH1 in LSCC remains unknown and needs to be examined. Increasing DACH1 expression is reported to bind to AP-1 and NF-kB sites to suppress IL-8 in cellular migration, and its carboxyl terminus binds to TCERG1 FF2 domain to repress transcription through DNA binding and YB-1C-terminus and DNA Binding Domain (DBD) to inhibit the development of breast cancer [15, 27, 28]. In our study, DACH1 suppressed the function of Hep-2 cells. DACH1 blocked both FOXM1 and FOXC2 to inhibit cell invasion, migration and cell cycle [5, 9]. C-Jun, as a component of AP-1 transcription factor complex, is inhibited by DACH1 to repress DNA synthesis and Cyclin D1 [29–31]. TIC or CSC is a stem cell that facilitates tumor initiation, however, re-expression of DACH1 decreased the tumor and mammosphere formation, reduced the CD44high/CD24low proportion in BTIC, and repressed the expression of Sox2, Oct4, Nanog, KLF4 and c-Myc [5, 32]. More recently, through Artificial Neural Networks (ANNs), DACH1 was shown as the biomarker that binds to ERа, and PELP1 is the target gene of DACH1 in ERа signaling [20, 33].

DACH1 is highly expressed in the nuclei, and is a new suppressor gene located on chromosome 13q22 [6]. DACH1 contains two domains: DachBOX-N and DachBOX-C, the former associates with SKI/SNO proteins (DS), and binds to HDAC3 and SIX6; while the latter binds to UBC9 [34]. DACH1 can block cell cycle progression and synergistic action with p53 protein and repress CXCL5 through protein-protein association in lung cancer and NSCLC [4, 35]. In vitro studies have shown that DACH1 negatively regulates colorectal cancer via Wnt pathway and malignant peripheral nerve sheath tumors through RAS signaling [19, 36]. Furthermore, DACH1 binds Smad4 and NCoR to repress TGF-β signaling [37], enhances chemosensitivity by inducing the expression of P21 [18], and regulates hormone levels in breast and prostate cancers [33, 38]. DACH1 responsive element (DRE) competes with FOXA-responsive element for binding sites, hence DACH1 attenuates FOX signaling [9, 28]. CCK8 and transwell assays showed that DACH1 inhibited cell proliferation and invasion in vitro, and its expression is lower in stage III-IV than in stage I-II. Therefore, DACH1 can inhibit cell invasion and metastasis, and loss of its function can facilitate the development of oncogenes.

DACH1 is thought to be an effective therapeutic target to suppress the invasion and growth of many tumors [39, 40]. Although surgery and radiation are the main treatments, the sequential treatment is not yet perfected and tumor cell recurrence cannot be controlled. Our study showed lower DACH1 in LSCC tissues than in normal tissues. Although over-expression of DACH1 suppressed cell proliferation and invasion, the underlying mechanisms need further study.

## Conclusion

In summary, this is the first report on the significance of DACH1 in LSCC. The expression of DACH1 was lower in LSCC than in adjacent normal tissues, and decreased expression of DACH1 was found in the tumors upraglottic tumor, lymph node metastases, T3–4 stage and advanced clinical stage. Positive regulation of DACH1 could suppress proliferation and invasion, and induce apoptosis of Hep-2 cells. Therefore, DACH1 may be a new therapeutic target to improve the prognosis of LSCC.

## Abbreviations

DAC: Dachshund
EMT: Epithelial-mesenchymal transition
LSCC: laryngeal squamous cell carcinoma
RDGN: Retinal Determination Gene Network
